# High levels of interleukin-6 are associated with final infarct size and adverse clinical events in patients with STEMI

**DOI:** 10.1136/openhrt-2021-001869

**Published:** 2021-12-21

**Authors:** Ingvild Maria Tøllefsen, Christian Shetelig, Ingebjørg Seljeflot, Jan Eritsland, Pavel Hoffmann, Geir Øystein Andersen

**Affiliations:** 1 Department of Cardiology, Oslo University Hospital Ulleval, Oslo, Norway; 2 Center for Clinical Heart Research, Department of Cardiology, Oslo University Hospital Ullevaal, Oslo, Norway; 3 Institute of Clinical Medicine, University of Oslo Faculty of Medicine, Oslo, Norway; 4 Section of Interventional Cardiology, Oslo universitetssykehus Ulleval, Oslo, Norway

**Keywords:** acute coronary syndrome, myocardial infarction, inflammation, MRI

## Abstract

**Objective:**

Inflammation has emerged as a new treatment target in patients with coronary artery disease and inflammation seems to play an important role in ischaemia/reperfusion injury that follows ST-elevation myocardial infarction (STEMI). We aimed to explore the role of acute and sustained interleukin 6 (IL-6) signalling, including soluble IL-6 receptor (IL-6R), with regard to infarct size, adverse remodelling and future cardiovascular events in patients with STEMI.

**Methods:**

We included 269 patients with first-time STEMI, symptom duration <6 hours and treated with percutaneous coronary intervention. Blood sampling and cardiac MRI were performed in the acute phase and after 4 months. Clinical events and all-cause mortality were registered during 12-month and 70-month follow-up, respectively.

**Results:**

IL-6 levels above median at all sampling points were significantly associated with increased infarct size and reduced left ventricular ejection fraction (LVEF). IL-6 levels in the highest quartile were at all sampling points associated with an increased risk of having an adverse clinical event during the first 12 months and with long-term all-cause mortality. IL-6R was not associated with infarct size, LVEF, myocardial salvage or long-term all-cause mortality.

**Conclusion:**

Acute and sustained elevation of IL-6 measured 4 months after STEMI were associated with larger infarct size, reduced LVEF and adverse clinical events including all-cause mortality. The results add important information to the sustained role of inflammation in patients with STEMI and IL-6 as a potential target for long-term intervention.

**Trial registration number:**

NCT00922675.

**Key questions**

**What is already known about this subject?**

- Atherosclerosis and acute coronary syndrome is known to be a product of both lipoprotein accumulation and inflammation.
- Inflammation seems to play an important role in ischaemia/reperfusion injury that follows ST-elevation myocardial infarction (STEMI).

**What does this study add?**

- High levels of circulating interleukin 6 (IL-6) measured early during reperfused STEMI were associated with future clinical events including long-term all-cause mortality.
- High levels of circulating IL-6 measured early during reperfused STEMI were associated with microvascular obstruction, large infarct size and reduced left ventricular ejection fraction after 4 months.
- High levels of IL-6 measured in a stable phase 4 months after STEMI were associated with both large infarct size and adverse prognosis.
- Circulating levels of soluble IL-6 receptor were not associated with infarct size or future clinical events.

**How might this impact on clinical practice?**

- Levels of IL-6 might be a marker of cardiovascular risk in patients with acute STEMI.
- The results add information to the role of IL-6 in myocardial injury and might select patients for anti-inflammatory therapy.

## Introduction

Inflammation is essential in all stages of coronary artery disease. Epidemiological studies have shown that an increased inflammatory response is associated with myocardial injury and worse prognosis in acute coronary syndromes,^1 2^ but the causality of inflammatory biomarkers are mainly unknown. During the past decades, studies have examined different pathways of inflammation in patients with cardiovascular disease (CVD), including the proinflammatory cytokines interleukin (IL)-1, IL-6 and IL-8.^1 3 4^ Targeted therapy against IL-1 in patients with CVD has been explored based on the role of IL-1 as an important regulator of downstream cytokine activation, including IL-6. Long-term IL-1β inhibition in the Canakinumab Anti-Inflammatory Thrombosis Outcomes Study demonstrated a reduction in major adverse cardiovascular events.^3^ The humanised anti-IL-6 receptor (IL-6R) antibody tocilizumab was studied in patients with non-STEMI, demonstrating reduced inflammation and troponin T levels^5^ and very recently in STEMI where a single infusion of tocilizumab during the acute percutaneous coronary intervention (PCI) was shown to increase myocardial salvage.^6^ In addition, soluble cytokine receptors regulate the activity of cytokine signalling. IL-6 can interact both with the membrane bound IL-6R (classical IL-6 signalling) or by binding to the soluble IL-6R (IL-6 trans-signalling).^7^ The IL-6/soluble IL-6R complex can bind to all cells in the body, regardless of membrane IL-6Rs, by binding to the transmembrane gp130 protein which is expressed in all cells.^8^ The temporal profiles of IL-6 and soluble IL-6R in patients with STEMI have previously been reported by Groot *et al*.^9^ They demonstrated an association between IL-6 and infarct size and left ventricular ejection fraction (LVEF), respectively. No association was found between IL-6 and reinfarction or mortality, but the event rate was very low.^9^

The objectives of the present study were to further explore IL-6 signalling during acute myocardial infarction (MI) by studying the potential role of acute and sustained IL-6 and soluble IL-6R elevation up to 4 months post-MI. The aims of the study were then to: (1) study possible associations between circulating IL-6 and IL-6R measured during acute STEMI and final infarct size, myocardial function, left ventricular (LV) remodelling and future cardiovascular events, (2) study the potential role of sustained IL-6 and IL-6R levels in the chronic post-MI patients with regard to future clinical events.

## Methods

### Study population

The present study was a substudy of the Post-conditioning in ST-elevation Myocardial Infarction (POSTEMI) trial including 269 of the 272 originally included patients. POSTEMI was a prospective, randomised, single-centre, open label clinical trial in which the possible cardio protective strategy ischaemic post-conditioning was investigated. The study has previously been described in details.^10 11^ Briefly, stable patients with first time STEMI and symptom duration <6 hours were included between 12 January 2009 and 25 August 2012 at Oslo University Hospital Ullevaal, Norway. Patients were excluded if they had ongoing treatment of angina pectoris before index infarction, previous MI or coronary artery bypass surgery, renal failure (creatinine ≥200 µmol/L), cardiogenic shock or contraindications to cardiac magnetic resonance (CMR) imaging. Primary PCI was performed in all patients, and they were randomised 1:1 to either post-conditioning or control after angiographic demonstration of a proximal occlusion of the infarct-related artery followed by successful reperfusion. Occlusion of the proximal or middle part of one of the three main coronary vessels (Thrombolysis In Myocardial Infarction (TIMI) flow 0–1) with no or minimal collateral flow to the ischaemic myocardium and successful reperfusion after the first balloon inflation (TIMI flow 2–3) had to be demonstrated angiographically before 1:1 randomisation either to the intervention group or to the control group. The control group was treated according to standard procedures.

### Laboratory analysis and methods

Blood samples were collected before PCI (median 2.8 hours after symptom onset) and immediately after PCI, at day 1 (median 18.3 hours after PCI) and at 4-month follow-up. Serum was prepared by centrifugation within 1 hour at 2.500 g for 10 min and stored at 80°C until analyses. Levels of IL-6 and IL-6R were analysed by enzyme immunoassays (ELISA), R&D Systems Europe, Abingdon, Oxon, UK. Interassay coefficients of variations for IL-6 and IL-6R were 5.2% and 3.3%, respectively. Further, in this article we refer to the soluble IL-6R when we describe results from analyses of the IL-6R. Routine blood samples included troponin-T and N-terminal pro-B-type natriuretic peptide (NT-proBNP), analysed by Elecsys 2010 (Roche Diagnostics, Mannheim, Germany) and C reactive protein (CRP), creatinine, haemoglobin, cholesterol, glucose and HbA1c, analysed by conventional laboratory assays. In the further analyses, we have used the peak levels of troponin T and CRP during the index hospitalisation.

### CMR protocol

The CMR protocol has been published in detail previously.^11^ Briefly, CMR was performed in the acute phase at median time of 2 days after admission (n=240) and repeated at 4-month follow-up (n=249). Images were obtained by a 1.5 T scanner (Philips Intera, release 11 or Philips Achieva, release 3.2, Philips Medical Systems Best, the Netherlands). Analyses were performed on an extended MR Work Space (Philips). Short axis images of the LV were acquired for volume analysis (ie, indexed for body surface area), and indexed LV end-diastolic volume (LVEDVi), indexed LV end-systolic volume and LVEF were calculated). Area at risk was analysed in T2-weighted short axis images. Late gadolinium enhancement imaging was used for analysing infarct size and microvascular obstruction (MVO). MVO was defined as the dark area within the hyperdense area in the infarcted myocardium. Estimation of myocardial salvage index (%) was measured by analysis of area at risk in the acute stage and final infarct size after 4 months; [(area at risk–infarct size at 4 months)/area at risk]×100.

### Clinical follow-up, adverse events and all-cause mortality

Follow-up and registration of adverse clinical events was performed 12 months after the index hospitalisation. A composite of clinical adverse events was defined as all-cause mortality, MI, stroke, rehospitalisation for heart failure or unscheduled revascularisation ≥3 months after the index event. In addition, long-term data on all-cause mortality were obtained from medical records after median 70-month follow-up.

### Statistical analyses

IL-6 and IL-6R were analysed both as continuous variables and grouped variables. Based on plots of the associations between quartiles (Q) of IL-6 and final infarct size, LVEF, myocardial salvage, change in LVEDVi and MVO, the median value was identified as a cut-off values for dichotomising IL-6 into high (≥median) and low (<median) levels for the CMR outcomes (online supplemental figure 1). The same cut-off value was used for IL-6R. Mann-Whitney U test and Kruskal-Wallis test were used for group comparisons of continuous variables, whereas categorical variables were analysed using χ^2^ test. Univariable and multivariable linear regression analyses were used to assess associations between IL-6 and final infarct size. Based on plots of the associations between Qs of IL-6 and adverse clinical events, Q4 (the 75th percentile) was identified as a cut-off value for dichotomising IL-6 into high (Q4) and low (Q1-3) levels. The same cut-off value was used for IL-6R. Kaplan-Meier curves with log-rank test and proportional hazard (Cox) regression were used to determine possible associations between IL-6 and IL-6R, and all-cause mortality. Unadjusted and adjusted OR for experiencing a composite endpoint during the first year was determined using logistic regression analyses. As a result of skewness, the following continuous variables were logarithmically transformed with the natural logarithm (ln): IL-6 and troponin T, CRP and NT-proBNP. The dynamic TIMI risk score is a composite risk score validated for estimation of 1-year mortality at the time of hospital discharge in patients with STEMI, and comprises baseline TIMI risk score (ie, age, presence of diabetes, hypertension or angina, systolic blood pressure <100 mm Hg, heart rate >100, Killip class II to IV, weight <67 kg, anterior infarction or left bundle branch block and time from symptom onset to PCI >4 hours) and index hospital events (ie, recurrent MI, stroke, major bleeding, shock, arrhythmia and renal failure).^12^ A two-sided p value <0.05 was considered statistically significant. All analyses were performed by IBM SPSS software, V.26.0 (SPSS).

## Results

### Temporal profile of IL-6 and IL-6R

In total, 269 patients with STEMI were included in this study, and blood samples for analyses of IL-6 and IL-6R at all time-points during the acute MI were available in 258 patients. Baseline characteristics according to levels of IL-6 (median 28.6 pg/mL) and IL-6R (median 33.5 ng/mL) at day 1 are presented in table 1.

**Table 1:** Characteristics of the study population as a whole and according to interleukin 6 (IL-6) levels and interleukin 6 receptor (IL-6R) (above or below median value) measured at day 1

	All patients	IL-6<median	IL-6>median	IL-6R<median	IL-6R>median
	N=258	(≤28.6 pg/mL)	(>28.6 pg/mL)	(≤33.5 ng/mL)	(>33.5 ng/mL)
**Baseline characteristics**					
Age (years)	60 (53–67)	57 (52–64)	62 (55–69)**	60 (53–66)	62 (52–68)
Male gender	212 (82.2%)	107 (82.9%)	106 (82.8%)	113 (85.0%)	100 (80.6%)
Body mass index (kg/m^2^)	26.6 (24.4–29.2)	27.1 (24.5–29.4)	26.3 (24.2–28.9)	26.3 (24.2–29.0)	27.1 (24.9–29.3)
Hypertension	72 (27.9%)	30 (23.3%)	41 (32.0%)	38 (28.6%)	33 (26.6%)
Hypercholesterolaemia	25 (9.7%)	15 (11.6%)	9 (7.0%)	15 (11.3%)	9 (7.3%)
Diabetes mellitus	16 (6.2%)	9 (7.0%)	7 (5.5%)	9 (6.8%)	7 (5.6%)
Current smoker	130 (50.4%)	65 (50.4%)	65 (50.8%)	68 (51.1%)	62 (50.0%)
**Clinical characteristics**					
Time from symptom to PCI (min)	187 (125–265)	172 (118–249)	197 (129–282)	190 (119–272)	184 (129–265)
Anterior MI†	126 (48.8%)	53 (41.1%)	73 (57.0%)*	65 (48.9%)	63 (49.2%)
Ischaemic post-conditioning‡	129 (50.0%)	64 (49.6%)	64 (50.0%)	67 (50.4%)	61 (49.2%)
**Biochemical analyses**					
Peak troponin T (ng/L)	5919 (3319–10463)	4900 (2693–7751)	7487 (4736–13061)***	6260 (3713–11244)	5174 (2628–9882)
Peak CRP (mg/L)	20 (8–52)	11 (5–23)	41 (16–84)***	20 (8–48)	18 (7–53)
Admission NT-proBNP (pmol/L)	9 (5–22)	8 (4–20)	12 (5–25)*	8 (4–21)	11 (5–23)
Admission creatinine (μmol/L)	70 (62–81)	70 (61–81)	72 (63–81)	68 (61–81)	73 (64–81)
Admission haemoglobin (g/dL)	13.4 (12.4–14.4)	13.3 (12.3–14.5)	13.6 (12.4–14.4)	13.3 (12.3–14.3)	13.6 (12.4–14.6)
Admission cholesterol (mmol/L)	5.1 (4.5–6.0)	5.2 (4.6–6.1)	5.1 (4.4–5.8)	5.2 (4.6–5.9)	5.1 (4.5–6.0)
Admission glucose (mmol/L)	8.0 (6.8–9.3)	7.7 (6.5–8.9)	8.1 (6.9–9.5)*	8.0 (6.8–9.6)	7.8 (6.8–9.6)
HbA1c (%)	6.0 (5.8–6.2)	6.0 (5.7–6.2)	6.0 (5.8–6.2)	6.0 (5.8–6.2)	6.0 (5.7–6.2)

Data are presented as median, number (percentage) and range. IL-6 and IL-6R was measured at day 1 (median 18.3 hours after PCI).

Significant values are highlighted in bold.

*p<0.05, **p<0.01, ***p<0.001 for comparison between high and low IL-6 and IL-6R levels (>/≤median).

†Infarct localisation—anterior myocardial infarction (MI) versus inferior or posterior MI.

‡Treated with ischaemic post-conditioning.

CRP, C reactive protein; NT-proBNP, N-terminal pro-B-type natriuretic peptide; PCI, percutaneous coronary intervention.

**Figure 1:**
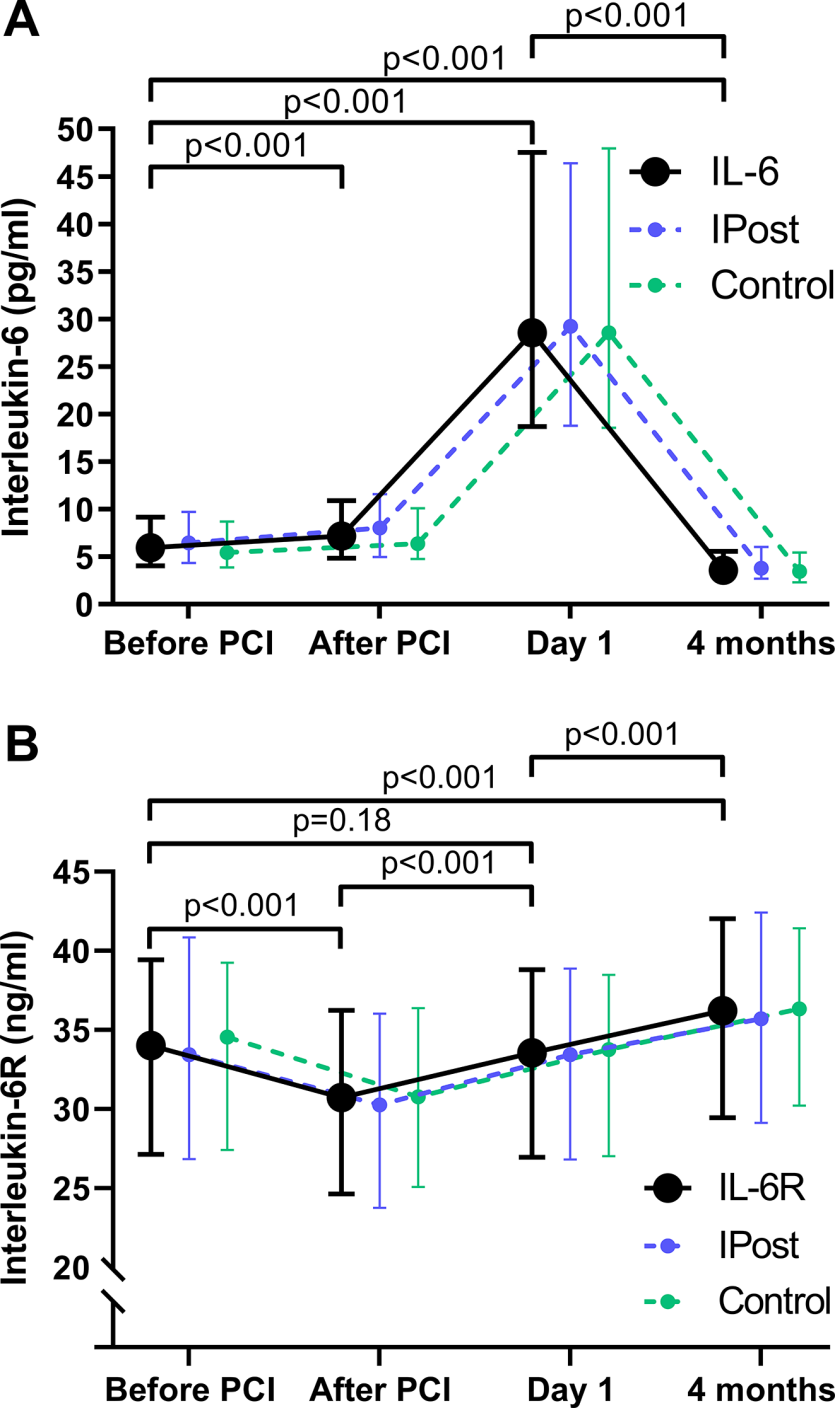
Temporal profile of IL-6 and IL-6R during ST-elevation myocardial infarction. IL-6 (A) and IL-6R (B) levels were measured before and immediately after the PCI procedure, at day 1 (median 18.3 hours after PCI), and at 4-month follow-up. Data are presented as median value (circles) with 25th and 75th percentile (whiskers) in all patients (black line), patients treated with ischaemic post-conditioning (dashed blue line) and patients treated with conventional PCI (dashed green line). IL, interleukin; IPost, ischaemic post-conditioning; PCI, percutaneous coronary intervention.

Figure 1 illustrates the temporal profiles of IL-6 (A) and IL-6R (B). Circulating IL-6 levels increased from admission to day 1, followed by a subsequent decrease at 4-month follow-up to levels below levels at admission (p<0.001) (figure 1A). IL-6R levels decreased during PCI, returning to similar levels as before PCI at day 1, and levels at 4-month follow-up was significantly higher than admission levels (figure 1B).

The ischaemic post-conditioning procedure was performed in an identical proportion of patients when comparing IL-6 and IL-6R levels above or below median value (table 1).

There were similar temporal profiles of IL-6 and IL6-R in patients randomised to the ischaemic post-conditioning procedure compared with conventional PCI (figure 1), and in further analyses the study population was analysed as a whole.

### Association between IL-6 levels and myocardial injury and function

Significantly higher levels of peak troponin T, peak CRP, admission NT-proBNP and admission glucose were found in patients with high levels of IL-6 (≥median) versus low levels at day 1 (table 1). High levels of IL-6 measured at all sampling points including 4 months were significantly associated with larger infarct size measured by CMR (table 2). High levels of IL-6 were also significantly associated with reduced LVEF both in the acute phase and after 4 months (table 2). In addition, patients with high IL-6 levels at day 1 had a higher frequency of MVO and reduced myocardial salvage index compared with patients with lower levels (table 2). Changes in LVEDVi from the acute phase to 4 months were significantly higher in patients with high levels of IL-6 measured at all time-points during the acute MI (table 2). In univariable linear regression analyses, high levels of IL-6 measured at day 1 was significantly associated with final infarct size assessed by CMR at 4 months (unstandardised β (β): 4.7; 95% CI 3.3 to 6.1; p<0.001). High levels of IL-6 at day 1 remained associated with final infarct size after adjustment for clinical (β: 3.7; 95% CI 2.4 to 5.0; p<0.001) and biochemical variables (β: 1.5; 95% CI 0.6 to 2.6; p=0.002) in multivariable linear regression analyses (table 3).

**Table 2:** Myocardial injury and function measured by CMR according to IL-6 values

	All patients	Before PCI			After PCI			Day 1			4 months		
		IL-6<median	IL-6>median	P value	IL-6<median	IL-6>median	P value	IL-6<median	IL-6>median	P value	IL-6<median	IL-6>median	P value
**CMR in acute phase**													
Infarct size (% of LV mass)	17.3	15.5	19.0	**0.02**	15.4	19.2	**0.04**	13.5	23.3	**<0.001**			
LV ejection fraction (%)	51	54	49	**<0.001**	53	49	**<0.001**	54	47	**<0.001**			
Presence of MVO	49.8%	47%	54%	0.23	46%	53%	0.29	37%	65%	**<0.001**			
**CMR after 4 months**													
Infarct size (% of LV mass)	13.9	12.1	16.1	**0.01**	13.1	16.1	**0.03**	11.0	17.2	**<0.001**	13.5	15.6	**0.02**
Myocardial Salvage (%)	51.9	51.9	51.5	0.60	51.9	52.1	0.56	58.1	44.7	**<0.001**	53.8	50.3	0.47
LV ejection fraction (%)	55	58	53	**0.003**	58	54	**0.005**	59	53	**<0.001**	58	53	**0.004**
∆EDVi (mL/m^2^)	4.5	2.3	6.2	**0.04**	2.2	6.2	**0.04**	1.0	8.1	**0.001**	4.5	4.1	0.41

Data are presented as median or percentage. IL-6 was measured before and immediately after the PCI-procedure, at day 1 (median 18.3 hours after PCI) and at 4-month follow-up in 261 patients with ST-elevation myocardial infarction.

Significant values are highlighted in bold.

CMR, cardiac MRI; ∆EDVi, change in end-diastolic volume (indexed for BSA) of LV from the acute phase to 4 months; IL, interleukin; LV, left ventricle; MVO, microvascular obstruction; PCI, percutaneous coronary intervention.

**Table 3:** Univariable and multivariable linear regression analyses of the associations between IL-6 measured at day 1 and final infarct size assessed by cardiac MRI at 4 months

Variable	β	95% CI	P value
**Univariable**			
IL-6 (ln), per SD	4.7	3.3 to 6.1	<0.001
Age	0.01	−0.1 to 0.1	0.93
Male sex	3.8	0 to 7.6	0.05
Time from symptom to PCI (per hour)	1.0	0.04 to 2.0	0.04
Anterior MI^a^	10.2	7.5 to 12.9	<0.001
Peak troponin T (ln), per SD	9.1	8.1 to 10.0	<0.001
**Multivariable model 1**			
IL-6 (ln), per SD	3.7	2.4 to 5.0	<0.001
Time from symptom to PCI (per hour)	0.9	0.06 to 1.8	0.04
Anterior MI	9.1	6.5 to 11.7	<0.001
**Multivariable model 2**			
IL-6 (ln), per SD	1.5	0.6, 2.6	0.002
Anterior MI	4.4	2.4, 6.4	<0.001
Peak troponin T (ln), per SD	7.8	6.7, 8.8	<0.001

Model 1: IL-6, age, gender, time from symptom to PCI, anterior MI. Model 2: model 1+peak troponin T. ^a^Infarct localisation—anterior myocardial infarction (MI) versus inferior or posterior MI.

IL, interleukin; ln, natural logarithm; PCI, percutaneous coronary intervention; β, unstandardised β.

### Association between IL-6R levels and myocardial injury and function

There were no significant associations between IL-6R levels and baseline characteristics, clinical characteristics or biochemical variables (table 1). IL-6R measured during hospitalisation was not associated with infarct size, LVEF or myocardial salvage. However, IL-6R measured at all sampling points during hospitalisation was modestly associated with LV remodelling measured as change in LVEDVi, also after adjustment for relevant clinical covariates in a multivariate linear regression model (online supplemental table 1).

### Adverse clinical events during 12-month follow-up and long-term all-cause mortality

A composite of adverse clinical events, occurred in 20 patients (7%) during 12-month follow-up and 26 patients (10%) died during 70-month follow-up.

**Figure 2:**
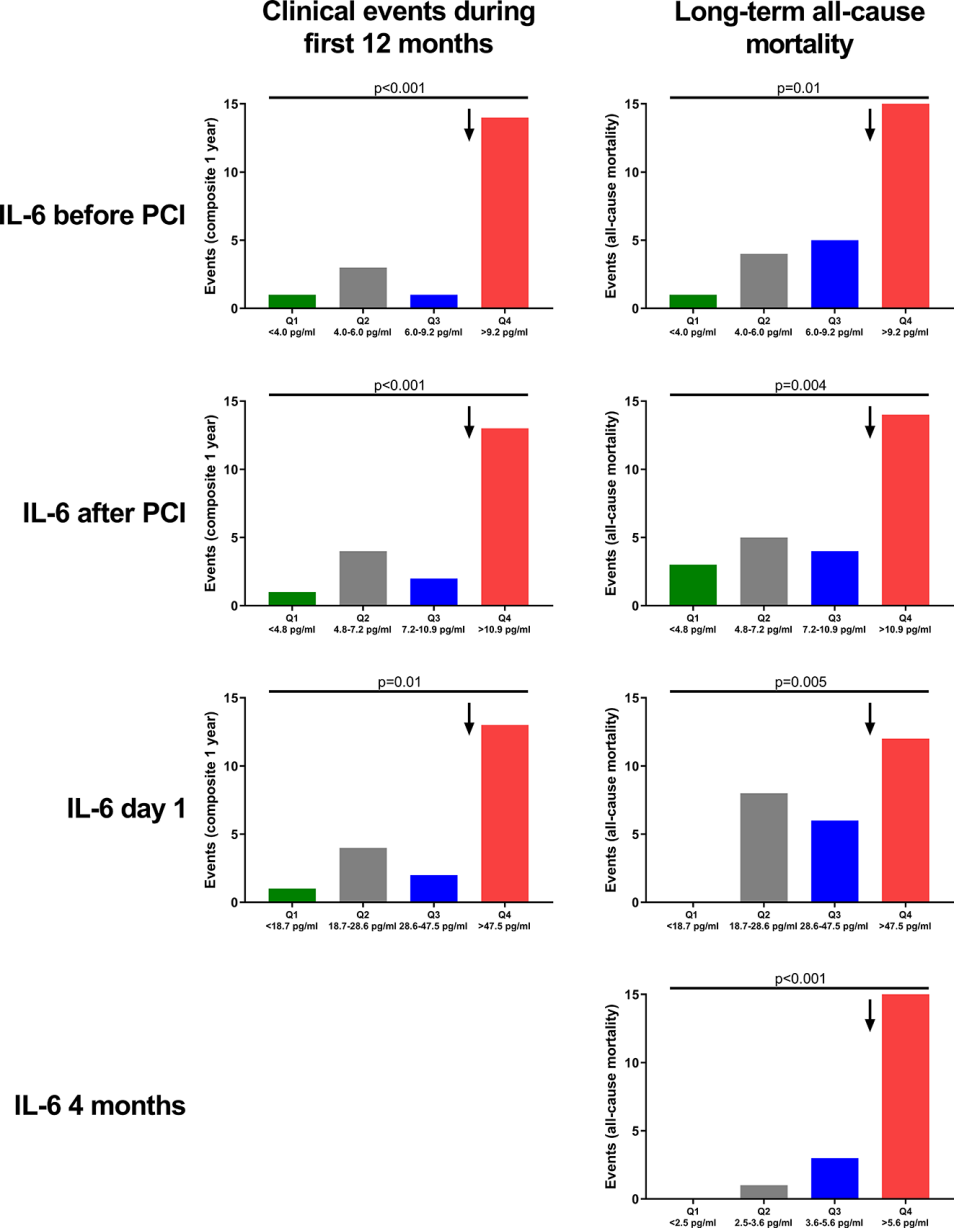
Adverse clinical events according to quartiles of IL-6 levels. IL-6 was measured before and immediately after PCI, at day 1 (median 18.3 hours after PCI), and at 4-month follow-up. Clinical events were registered during the first 12 months and were defined as all-cause mortality, myocardial infarction, unscheduled revascularisation ≥3 months after the index infarction, rehospitalisation for heart failure or stroke. Long-term all-cause mortality was registered during median 70 months of follow-up. Data presented as absolute numbers. Arrows indicate the 75th percentile. P values for difference across quartiles of IL-6. IL, interleukin; PCI, percutaneous coronary intervention; Q, Quartile.

**Figure 3:**
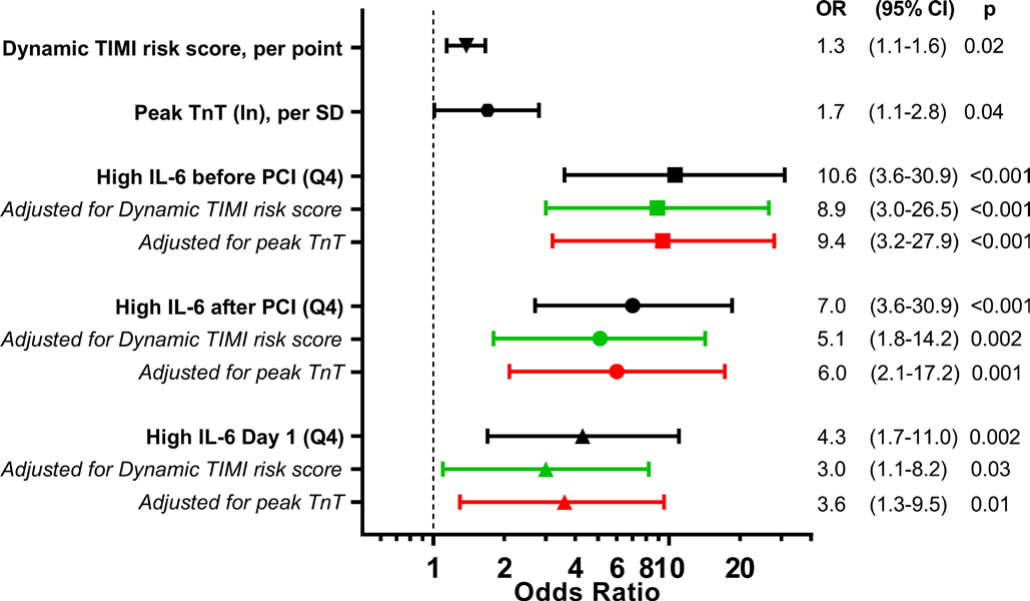
Unadjusted and adjusted ORs for experiencing a composite endpoint during the first 12 months after ST-elevation myocardial infarction when having high IL-6 levels (>75th percentile). IL-6 was measured before and immediately after PCI, and at day 1 (median 18.3 hours after PCI). The composite endpoint was defined as all-cause mortality, myocardial infarction, unscheduled revascularization ≥3 months after the index infarction, rehospitalisation for heart failure, or stroke. IL, interleukin; PCI, percutaneous coronary intervention; Q4, 4th quartile; TIMI, Thrombolysis In Myocardial Infarction; TnT, Troponin T.

**Figure 4:**
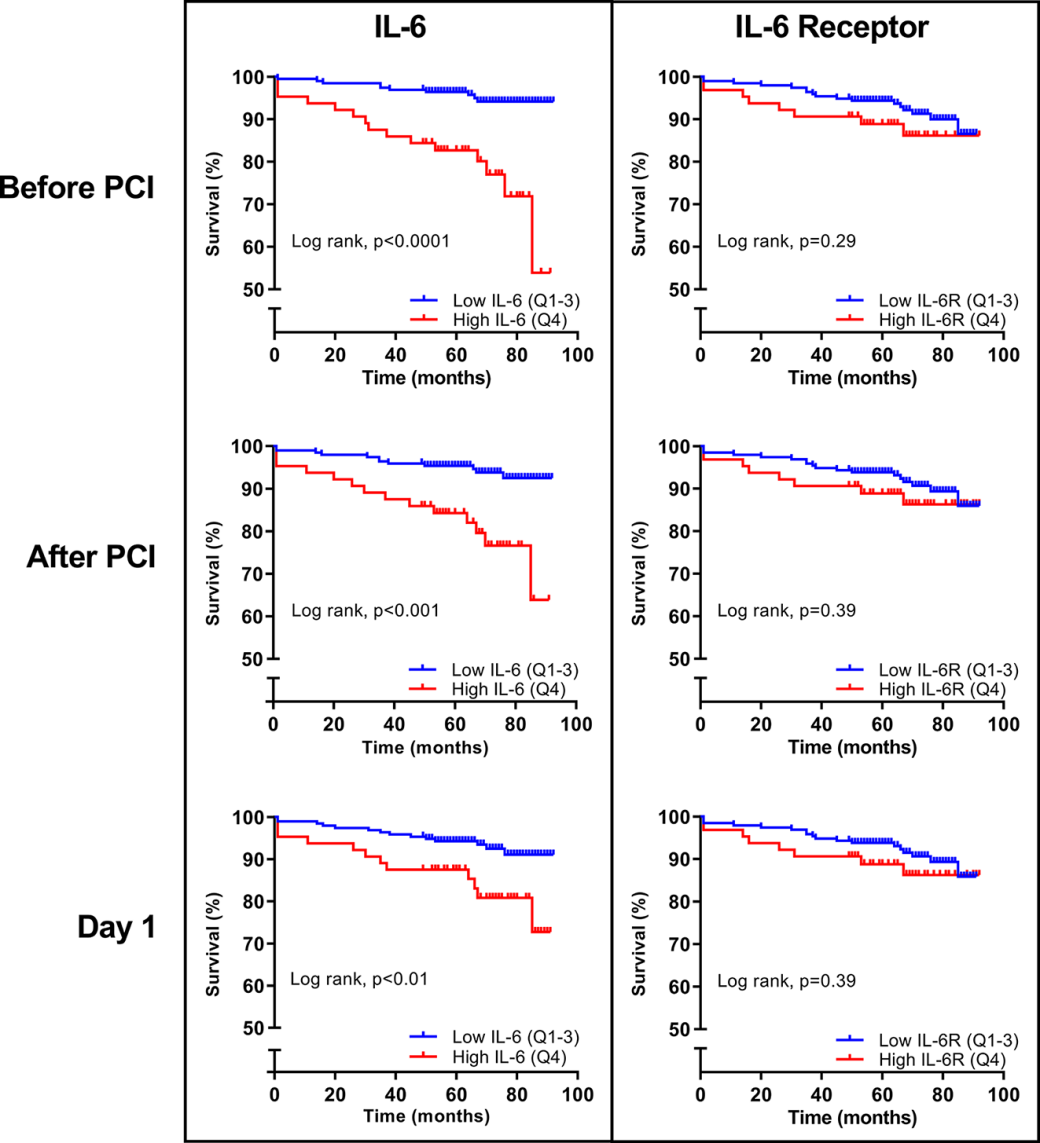
Kaplan-Meier curves of all-cause mortality according to high or low IL-6 and IL-6R (>/≤75th percentile) measured during hospitalisation with ST-elevation myocardial infarction. IL-6 and IL-6R were measured before and immediately after PCI and at day 1 (median 18.3 hours after PCI). All-cause mortality was registered during median 70 months of follow-up. IL, interleukin; IL-6R, IL-6 receptor; PCI, percutaneous coronary intervention; Q, quartile.

**Figure 5:**
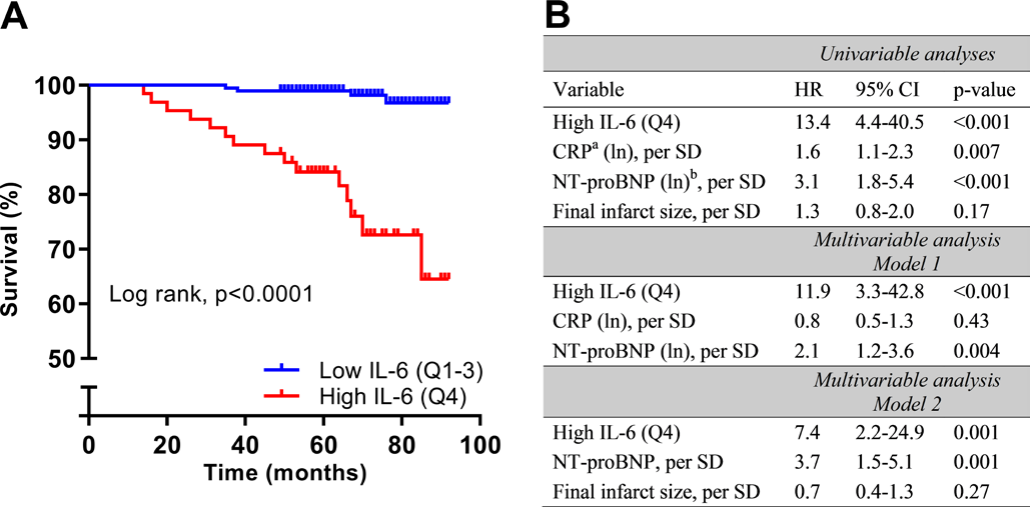
Risk of all-cause mortality according to interleukin 6 (IL-6) levels measured 4 months after ST-elevation myocardial infarction (STEMI). (A) Kaplan-Meier plot of all-cause mortality during long-term follow-up (median 70 months) according to high or low (>/<75th percentile) IL-6 levels measured 4 months after STEMI. (B) Univariable and multivariable HRs for long-term all-cause mortality when having high (>75th percentile) IL-6 levels 4 months after STEMI. Data are presented as HRs with 95% CI and p values obtained by Cox regression. IL-6, CRP, NT-proBNP and final infarct size were measured at 4-month follow-up. ^a^CRP, C reactive protein; ^b^NT-proBNP, N-terminal pro-B-type natriuretic peptide.

High levels of IL-6 (Q4) in the acute phase remained associated with an increased risk of experiencing a composite endpoint during the first 12 months after STEMI also after adjustment for dynamic TIMI risk score and peak troponin T levels (figures 2 and 3). Patients with high levels (Q4) of IL-6 measured at all time-points in the acute phase had an increased risk of death during long-term follow-up (figure 4). Interestingly, this association remained 4 months after STEMI. Patients with IL-6 levels in the upper quartile, indicating a sustained inflammatory response, had increased risk of death during median 70 months of follow-up with an HR of 13.4 (95% CI 4.4 to 40.5; p<0.001). Patients with IL-6 levels in the upper quartile had increased risk of death also after adjustment in multivariate Cox regression analyses adjusting for CRP, NT-proBNP and final infarct size, measured 4 months after STEMI (figure 5). There were no significant associations between IL6-R and the composite primary endpoint and long-term all-cause mortality (figure 4).

## Discussion

In patients with first-time STEMI treated with PCI, high levels of IL-6 at all sampling points during hospitalisation were significantly associated with increased infarct size, reduced LVEF and an increased risk of experiencing a composite endpoint during the first 12 months after STEMI. Additionally, high levels of IL-6 at day 1 were associated with lower myocardial salvage, more frequent MVO and more pronounced adverse LV remodelling. Importantly, patients with sustained high levels of IL-6 4 months after STEMI, indicating residual inflammatory risk, had increased risk of death during 70-month follow-up. IL-6R measured during hospitalisation was significantly associated with LV remodelling measured as changes in LVEDVi, but was not associated with infarct size, LVEF, myocardial salvage or future adverse clinical events.

The temporal profile of IL-6 demonstrated increased levels at admission (compared with 4 months after STEMI) with high levels in blood sampled the following morning, median 18.3 hours after PCI. There was a significant, but minor rise in IL-6 levels during PCI. This is in agreement with previous studies on patients with acute MI demonstrating high levels of IL-6 during acute STEMI.^9 13 14^ Contrary to the curved time course of IL-6, the IL-6R time course was rather flat with decreased levels during the acute STEMI compared with 4 months later, again in agreement with Groot *et al*.^9^

We extend and confirm previous studies including our own demonstrating a close association between IL-6 and mycardial necrosis measured both as troponin release and as infarct size visualised by CMR.^9 14 15^ Interestingly, the association between IL-6 at day 1 and infarct size remained significant also after adjustment for troponin. The importance of inflammation was also demonstrated by the close association between IL-6 and LV function suggesting a role in adverse LV remodelling and potential development of heart failure. The demonstrated association between high IL-6 levels and higher frequency of MVO and reduced mycardial salvage, respectively, also indicate a role of inflammation in the ischaemia-reperfusion injury which play an important role in final infarct size development. The pathophysiology of ischaemia-reperfusion injury has not been fully elucidated, but inflammation has been shown to be involved.^16^

We did not find significant associations between IL-6R and neither troponin levels nor LVEF in our population which is in accordance with previous reports.^9 15^ Soluble IL-6R is largely generated by proteolytic cleavage of membrane bound IL-6R by metalloproteinases.^17^ The relative importance of soluble IL-6R and IL-6 has not been fully elucidated. Trans-signalling by circulating IL-6/IL-6R complex may be as important in mediating proinflammatory effects as the classical IL-6 signalling pathway which is mediated by binding of IL-6 to the membrane bound IL-6R.^8^ IL-6R inhibition by tocilizumab has recently been shown to reduce myocardial injury and increase myocardial salvage in patients with MI^5 6^ and IL-6 ligand inhibition by ziltivekimab was very recently reported to reduce biomarkers of inflammation and thrombosis relevant to atherosclerosis in patients with chronic kidney disease and elevated CRP.^18^ Tocilizumab seems to bind to both the membrane and soluble form of IL-6R and complete inhibition of IL-6 intracellular signalling pathways has been reported in cell cultures.^19^ We found a significant, but modest association between LVEDVi and IL-6R, but no associations were found with regard to infarct size or LVEF or other markers of impaired myocardial function.

A causal association between IL-6R signalling and atherosclerosis and coronary heart disease has been demonstrated in both human genetic studies and clinical studies on biomarkers.^20 21^ In patients with acute MI, both acute short-term inflammation and sustained low-grade inflammation may be important for short-term prognosis as well as risk of new cardiovascular events.

There has been somewhat conflicting results regarding IL-6 levels and prognosis in patients with STEMI. In the present study, high levels of IL-6 at all four sampling points were significantly associated with increased risk of long-term all-cause mortality, in accordance with some other studies.^1 2^ Other studies, including one from our own research group, however, found no association between IL-6 and new clinical events.^7 9^ The present study however, demonstrates a consistent association between IL-6 measured at all time-points and the risk of experiencing a composite of clinical events during the first 12 months after STEMI also after adjusting for troponin and dynamic TIMI risk score. We could not confirm previous findings of an association between IL-6R and long-term adverse events in patients with STEMI.^7^ The reason for this discrepancy is not known, but may involve differences in infarct size and level of inflammation. The present study included selected patients with larger infarct size compared with a previous study^7^ and the IL-6 levels were different (median 28.6 vs 18.8 pg/mL at day 1, respectively).

Since anti-inflammatory therapy is emerging as an option in both acute and chronic CVD, identifying patients at risk is important. Our study adds important information to the proposed hypothesis of patients with residual inflammatory risk.^20^ Very interestingly, patients with sustained high levels of IL-6 4 months after STEMI had increased risk of all-cause mortality during median 70 months of follow-up and this association remained after adjusting for NT-pro-BNP and final infarct size, possibly identifying patients which may benefit from long-term anti-inflammatory treatment.

### Study limitations

The main study, POSTEMI, was designed to study PCI with or without ischaemic post-conditioning in patients with STEMI, and information on baseline characteristics were sparse, and we did not have information on the specific cause of death. Another limitation is that blood samples were taken in the acute phase up to day 1 and then at 4 months, and we might have missed peak-values of IL-6 and IL-6R due to lack of repeated blood sampling. Chronic inflammatory disease was not an exclusion criterion in the POSTEMI study; however, only six of the included patients had chronic inflammatory diseases using anti-inflammatory medication suggesting that inclusion of these patients did not influence the results of this study. The POSTEMI study was not powered to study the effect of ischaemic post-conditioning on mortality. The present substudy had an explorative design. The demonstrated association between IL-6 and mortality must be interpreted with caution due to the low number of events.

## Conclusions

Patients with high IL-6 levels during the acute phase of STEMI had larger infarct size, reduced myocardial salvage, reduced LV function and worse clinical outcome than patients with lower levels of IL-6. High levels of IL-6 measured after 4 months were also associated with larger infarct size, reduced LVEF and increased long-term all-cause mortality. The results add important information to the role of IL-6 in myocardial injury in acute STEMI and levels of IL-6 might be a marker of cardiovascular risk and also select patients for anti-inflammatory therapy.

## Data Availability

Data are available upon reasonable request.
